# Rapid Immune Colloidal Gold Strip for Cetacean Meat Restraining Illegal Trade and Consumption: Implications for Conservation and Public Health

**DOI:** 10.1371/journal.pone.0060704

**Published:** 2013-03-28

**Authors:** Chieh Lo, Li-Te Chin, Chi-Shih Chu, Yu-Ting Wang, Kun-Wei Chan, Wei-Cheng Yang

**Affiliations:** 1 Department of Veterinary Medicine, National Chiayi University, Chiayi, Taiwan (R.O.C.); 2 Department of Microbiology, Immunology and Biopharmaceuticals, National Chiayi University, Chiayi, Taiwan (R.O.C.); Biodiversity Insitute of Ontario - University of Guelph, Canada

## Abstract

The consumption of cetacean meat is geographically common and often of undetermined sustainability. Besides, it can expose humans to contaminants and zoonotic pathogens. The illegality of possessing cetacean meat was likely under-reported in some countries due to lack of attention paid by the officials although DNA analysis of market products helped to show such practices. We developed two monoclonal antibodies against synthetic peptides of myoglobin (Mb) for constructing a rapid immune colloidal gold strip. Only cetacean Mb is capable of binding to both antibodies and presents positive signal while the Mb from other animals can bind only 1 of the antibodies and presents negative result. The strip for cetacean meat would be an applicable and cost-effective test for field inspectors and even the general public. It contributes to increase the reporting capacity and coverage of illegal cetacean meat possession, which has implications for global cetacean conservation and public health.

## Introduction

It is now obvious that human consumption of cetaceans is geographically widespread and taxonomically diverse [1]. Since cetaceans are the highest-level marine predators, heavy metal and toxin would accumulate in cetacean meat [2]. Endo et al. [3] reported high mercury (T-Hg) and methyl mercury (M-Hg) levels in cetacean meat products. The highest M-Hg, 26 µg/wet g, was found in a meat sample from a stripped dolphin. Taking only 4 g of that product would be in excess of the provisional tolerable weekly intake of M-Hg for an adult (1.6 µg/kg-bw/wk) [4]. The meat consumption of small cetaceans could cause health problems to the general population and high-risk group such as pregnant women [3]. Furthermore, when they handle or consume cetacean meat, humans may be infected with pathogens by direct or indirect contact [5]. An extensive range of zoonotic or potentially zoonotic parasites, fungi, bacteria and viruses have been discovered in cetaceans, and in some cases transmission of pathogens from cetaceans have resulted in human diseases [6]. It was suggested that the infectious agents as possible zoonotic pathogens associated with cetacean meat and products should be under particular focus [5].

For restraining cetacean meat from being trafficked or consumed, first we need to differentiate it from other meats. DNA-based PCR assay is currently available for cetacean identification [7], and it helped provide evidence of illegal international trade of cetacean meat in at least Hong Kong, Japan, Norway, the Philippines, Russia, South Korea, Taiwan, and the United States [1]. Although the method has advantages of great sensitivity and specificity, it requires the use of major laboratory equipment, long assay time, and trained analysts to conduct the assay. Therefore it is needed to have simple and reliable scientific methods for identification. Currently there is no rapid immunoassay, such as a strip test or enzyme-linked immunosorbent assay (ELISA) kit, capable of identifying cetacean meat. Polyclonal or monoclonal antibodies are essential in all immunoassays. Monoclonal antibodies (mAbs) are homogeneous populations of antibodies produced by cell-fusion hybridoma technology. They have determined biological activities, consistent specificity, and unlimited production capability [8]. In contrast, polyclonal antibodies (pAbs) generated using multiple animals will differ among immunized animals, and their avidity may change as they are harvested over time. Cetacean-specific mAbs can be used to construct inexpensive rapid field immunoassay test kits, enabling untrained inspectors to verify a cetacean meat sample. A protein abundant in muscle could be a good candidate for raising mAbs.

Mammals that can dive to depths greater than 100 m, such as cetaceans and seals, usually have muscles that contain high concentration of myoglobin (Mb), which enable aerobic metabolism to be maintained during breath hold and represent the key adaptation for diving [9], [10]. Mb is a single-chain globular protein (153 or 154 amino acids) composed by 8 alpha helices and a hydrophobic core including the heme (iron-containing porphyrin) prosthetic group in the center around which the remaining apoprotein folds [11]. The molecular weight of Mb is already known as 14–18 kDa (reviewed in [12]). Mb promotes transcellular oxygen diffusion and provides intracellular oxygen storage in the muscle tissues. The oxygen store in muscle is affected by the muscle mass and the concentration of Mb [10]. In manatee, the color of muscle from almost white to dark red depends on different concentrations of Mb [13]. The Mb concentration of wet muscle in beef, lamb, pork, and poultry are 8, 6, 2, and 1–3 mg/g, respectively [14]. In comparison with these domestic meats, cetacean meat contains higher concentration of Mb (20–70 mg/g) [9], [15]. Besides, the concentration of Mb in cetacean muscle varies from different locations and different species, which is related to the contraction strength of muscle, the need of persistent work, and the cetaceans’ habitat as well [15]. The interior of the muscles closest to the vertebrae such as *m. longissimus dorsi* showed a significantly higher mean Mb concentration than the exterior of the muscle [16].

Iwanami et al. [17] studied 15 representative amino acid sequences of cetacean Mb from Balaenopteridae, Physeteridae, Kogiidae, Delphinidae, Phocoenidae and Ziphidae by phylogenetic analysis and the sequences of Mb of human and bovine were used as outgroup. The result showed 124/153 amino acid residues among cetaceans were identical, leaving 29 informative positions. The authors suggested that the phylogenetic analysis of Mb amino acid sequences was at least valid for classification of cetaceans at the family level, indicating the high similarity of Mb among cetaceans. We further noticed 7 conserved regions in the amino acid sequences of cetaceans in Iwanami et al.’s work, which could be the candidate epitopes and provides an opportunity to develop the specific mAbs against Mb in cetaceans. Here we show a simple, reliable, and economic mAb-based scientific identification method of cetacean meat for field inspectors and even the general public that contributes to increase the reporting capacity and coverage of illegal cetacean meat possession.

## Materials and Methods

### Ethics Statement

The animal use protocol has been reviewed and approved by the Institutional Animal Care and Use Committee (IACUC) of National Chiayi University (Approval number 99022). For the care and use of laboratory animals, experiments were performed in accordance with international guidelines. No animals were sacrificed specifically for muscle sample collection. The research permit (100M-02.1-C-99) for the cetacean sample collection was provided by Council of Agriculture of Taiwan.

### Muscle samples from various animals and sample preparation

Muscle samples were collected from tuna, chicken, 6 species of terrestrial mammals, and 16 species of marine mammals (Table 1). The cetacean muscle samples were obtained from stranded individuals, fishery bycatch, and confiscation. DNA extraction, PCR and sequencing of the cetacean samples were performed as described previously [18]. The sequence analyses were conducted with the BLAST Search option in GenBank (http://www.ncbi.nlm.nih.gov/blast/Blast.cgi) and DNA surveillance (http://www.dna-surveillance.auckland.ac.nz) to confirm the species identity of cetaceans. Rabbit, rat, dog, and chicken muscle tissues were obtained from the carcasses of Animal Disease Diagnostic Center of National Chiayi University. The muscle sample of harbor seal (*Phoca vitulina*) was provided by Farglory Ocean Park. Samples of beef, pork, lamb, and tuna were purchased from a local supermarket. All samples were stored at −20°C until use. Three grams of frozen muscle sample of each animal was put into beakers pre-cooled at −20°C. The sample was homogenized with 10 mL phosphate buffered saline (PBS)(AMRESCO, Solon, OH, USA). The supernatants were collected from the homogenized sample by centrifugation at 10000 x g for 10 min at 4°C and were stored at −20°C until use.

**Table 1:** The species from which muscle was collected and tested in this study.

Cetacean species	Non-cetacean species
Common minke whale (*Balaenoptera acutorostrata*)	Cattle (*Bos Taurus*)
Omura’s whale (*Balaenoptera omurai*)	Goat (*Capra hircus*)
Pygmy sperm whale (*Kogia breviceps*)	Pig (*Sus scrofa*)
Dwarf sperm whale (*Kogia sima*)	Dog (*Canis lupus familiaris*)
Short-finned pilot whale (*Globicephala macrorhynchus*)	Rabbit (*Oryctolagus cuniculus*)
Melon-headed whale (*Peponocephala electra*)	Rat (*Rattus norvegicus*)
Pygmy killer whale (*Feresa attenuata*)	Chicken (*Gallus gallus*)
Pantropical spotted dolphin (*Stenella attenuata*)	Yellowfin tuna (*Thunnus albacares*)
Bottlenose dolphin (*Tursiops truncatus*)	Harbor seal (*Phoca vitulina*)
Bottlenose dolphin (*Tursiops aduncus*)	
Fraser’s dolphin (*Lagenodelphis hosei*)	
Indo-Pacific humpback dolphin (*Sousa chinensis*)	
Rough-toothed dolphin (*Steno bredanensis*)	
Risso’s dolphin (*Grampus griseus*)	
Finless porpoise (*Neophocaena phocaenoides*)	

### Peptide design, synthesis, immunization and splenic fusion

The Mb amino acid sequences obtained from GenBank, including 18 species of cetaceans from Balaenopteridae, Eschrichtiidae, Delphinidae, Phocoenidae and Kogiidae, 6 species of other mammals, 2 species of poultries, and 1 fish species, were aligned using MEGA 5.0 [19] (Table 2). The analysis focused at 5 antigenic reactive regions that were showed in the previous study [20]: region 1 (AKVEADVA, 15−22), region 2 (KASEDLK, 56−62), region 3 (ATKHKI, 94−99), region 4 (HVLHSRH, 113−119) and region 5 (KYKELGY, 145−151). According to the sequence analysis, candidate sequence fragments were synthesized and conjugated with carrier protein (OVA).

**Table 2:** Myoglobin sequences used in this study with respective GenBank accession numbers.

Species	Accession no.
Common minke whale (*Balaenoptera acutorostrata*)	P02179
Pygmy Bryde's whale (*Balaenoptera edeni*)	Q0KIY2
Humpback whale (*Megaptera novaeangliae*)	P02178
Gray whale (*Eschrichtius robustus*)	P02177
Sperm whale (*Physeter macrocephalus*)	P02185
Pygmy sperm whale (*Kogia breviceps*)	Q0KIY5
Dwarf sperm whale (*Kogia sima*)	P02184
Short-beaked common dolphin (*Delphinus delphis*)	P68276
Long-finned pilot whale (*Globicephala melas*)	P02174
Killer whale (*Orcinus orca*)	P02173
Melon-headed whale (*Peponocephala electra*)	Q0KIY3
Pantropical spotted dolphin (*Stenella attenuata*)	Q0KIY6
Bottlenose dolphin (*Tursiops truncatus*)	P68279
Harbor porpoise (*Phocoena phocoena*)	P68278
Amazon river dolphin (*Inia geoffrensis*)	P02181
Longman's beaked whale (*Indopacetus pacificus*)	Q0KIY9
Hubbs' beaked whale (*Mesoplodon carlhubbsi*)	P02183
Cuvier's beaked whale (*Ziphius cavirostris*)	P02182
Harbor seal (*Phoca vitulina*)	P68080
Cattle (*Bos Taurus*)	P02192
Goat (*Capra hircus*)	B7U9B5.3
Horse (*Equus caballus*)	P68082
Pig (*Sus scrofa*)	P02189
Dog (*Canis lupus familiaris*)	P63113
Chicken (*Gallus gallus*)	P02197
Ostrich (*Struthio camelus*)	P85077
Yellowfin tuna (*Thunnus albacares*)	P02205

Five female BALB/c mice were immunized subcutaneously with 0.1 mg/mouse of each synthetic peptide (immunogen) in PBS emulsified with an equal volume of complete Freund’s adjuvant followed by five subcutaneous booster injections with the same concentration of the immunogen emulsified with the equal volume of incomplete Freund’s adjuvant at 2-wk interval. Test sera from the mice were collected every 2 wk before next booster injection. The titer of the sera was determined by indirect ELISA coated with the free peptide for the first screening. The mouse displaying the highest titer would be injected with 0.1 mg of the immunogen 3 days prior to fusion. The procedure of hybridoma production was modified from Köhler and Milstein [21]. Briefly, murine myeloma cells F0 (sp2/0-Ag14) from commercial source (LTK BioLaboratories, Taiwan) were fused with the spleen cells from the immunized mice. The hybridomas supernatants were screened by indirect ELISA 14 days after fusion for testing the reactivity toward free peptide. The positive clones were selected, cultured twice with limiting dilution, screened by western blot and dot blot, and then expanded. The reactivity of the mAbs from hybridoma supernatants toward the muscle extracts from different species was tested by western blot and dot blot. The mAbs were purified from the ascites fluid of mice inoculated with the hybridoma cells by Protein G HP SpinTrap (GE Healthcare, Piscataway, NJ, USA). The isotypes of mAbs were determined by IsoStrip Mouse Monoclonal Antibody Isotyping Kit (Roche Diagnostics, Indianapolis, IN, USA) following manufacturer’s instructions.

### SDS–PAGE

Muscle extracts containing soluble proteins with different molecular weights were separated by SDS-PAGE following the method from Laemmli [22] with modifications. The stacking gel (15%) and separating gel (5%) were made in Mini Trans-Blot Cell (Bio-Rad, Hercules, CA, USA) for electrophoresis. The voltage was at 100 volts for 20 min first, and following-up with 120 volts for 40 min. Then the gel was stained with Coomassie brilliant blue for 30 min at room temperature and then destained with acetic acid solution (10%) for 1 h at room temperature. The gel destaining procedure was subsequently repeated at 4°C overnight.

### Western blot

The extracts were diluted 1∶5 (terrestrial animals and tuna) and 1∶50 (marine mammals) when using commercial rabbit anti-human Mb polyclonal antibody (Santa Cruz Biotechnology, Santa Cruz, CA, USA), and diluted 1∶1 (pig, rabbit, chicken and tuna), 1∶5 (cow, goat and dog), and 1∶25 (marine mammals) when using hybridoma supernatants. The soluble proteins of muscle extracts were transferred to nitrocellulose (NC) membranes (Whatman, Florham Park, NJ, USA) after they were separated by electrophoresis. The membrane was blocked with 5% skim milk in PBS containing 0.1% Tween 20 (PBST) for 1 h at room temperature, washed with PBST, and then incubated with the commercial antibody diluted 1∶2000 in 5% antibody blocker or hybridoma supernatant diluted 1∶3 in 5% antibody blocker solution (LTK BioLaboratories, Taoyuan, Taiwan) for 4°C overnight. After the washing step to remove the extra antibody with PBST, the membrane was incubated with goat anti-mouse IgG labeled with alkaline phosphatase diluted 1∶1250 in 5% antibody blocker solution for 1 h at room temperature. After washing, the membrane was soaked in the BCIP/NBT phosphatase substrate (contains 5-Bromo-4-chloro-3-indoxyl-phosphate at a concentration of 0.21 g/L and nitroblue tetrazolium at a concentration of 0.42 g/L in an organic base/Tris buffer) (KPL, Gaithersburg, MD, USA) within 10 to 20 min until the color development was observed. The staining reaction was stopped by washing the membrane with distilled water.

### Dot blot

The muscle extracts of different species were diluted 1∶5 (terrestrial animals and tuna) and 1∶25 (marine mammals) and blotted on NC membrane. The membrane was dried out in a laminar flow, blocked with 5% skim milk in PBST for 1 h at room temperature, washed with PBST, then incubated with the mAb supernatant diluted 1∶3 in 5% antibody blocker solution for 1 h at room temperature. Through the washing step to remove the extra antibody with PBST, the membrane was incubated with goat anti-mouse IgG labeled with alkaline phosphatase diluted 1∶1250 in 5% antibody blocker solution for 1 h at room temperature. After washing, the membrane was soaked in the BCIP/NBT phosphatase substrate within 10 to 20 min until the color development was observed. The staining reaction was stopped by washing the membrane with distilled water.

### Indirect ELISA

The Protein Detector ELISA Kit (KPL) was used for quantification of cross-reactivity of the purified mAbs toward muscle extracts. A 96-well ELISA plate (Nunc Immunoplate MaxiSorp; Thermo Fisher Scientific, Rochester, NY, USA) was coated with 100 µL of muscle extracts diluted 1∶25 in coating buffer at 4°C overnight and blocked with blocking/diluted buffer (1% bovine serum albumin in PBS) for 1 h at room temperature. One hundred microliters of the purified mAbs diluted 1∶2000 in blocking/diluted buffer were added to each well and incubated for 1 h at room temperature followed by further incubation of goat anti-mouse IgG conjugated with horseradish peroxidase (diluted 1∶200 in blocking/diluted buffer). The plate was washed 3 times with washing buffer (0.002 M imidazole buffered saline with 0.02% Tween 20) between each step. ABTS^®^ peroxidase substrate (100 µL/well) was added for 10 min until the color development and then the enzyme reaction was stopped by the ABTS^®^ peroxidase stop solution (100 µL/well). Absorbance was read at 450 nm using the Multiskan EX ELISA reader (Thermo Electron Corporation, Waltham, MA, USA).

### Preparation of colloidal gold-labeled mAb

Colloid gold (40 nm) solution (REGA biotechnology Inc., Taipei, Taiwan) was adjusted to pH 8.0 with 0.1 M potassium carbonate. The optimum concentration of purified detecting mAb (60 µg) was added to 10 mL colloid gold solution and incubated at room temperature for 10 min with gently mixing. The mixture was blocked with 2 mL of 5% bovine serum albumin (BSA; Gibco, Carlsbad, CA, USA) solution in phosphate buffered saline (PBS; pH 7.4, AMRESCO) at room temperature for 15 min with gently mixing and centrifuged at 10,000 x g at 4°C for 30 min. The gold pellets were suspended by PBST (containing 1% BSA and 0.1% Tween 20), and centrifuged and suspended for washing repeatedly. Final precipitates were suspended in 1 mL PBST (containing 1% BSA and 0.1% Tween 20) and stored at 4°C until used.

### Preparation of immune strip

**Figure 1:**
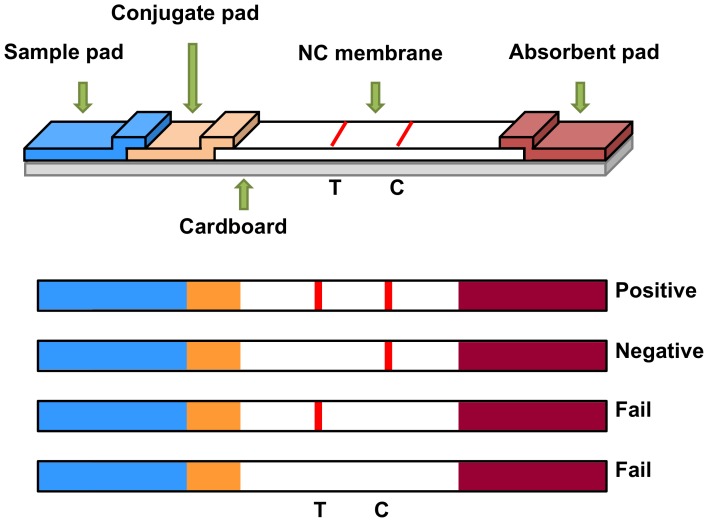
The preparation, construction and design of the immune strip. T: test zone. C: control zone.

The preparation, construction and design of the immune strip was described as follows and shown in Fig. 1. NC membranes, sample pads, conjugate pads and absorbent pads were all purchased from REGA biotechnology Inc.. The conjugate pad was saturated with colloid gold solution (labeled mAb) and then dried at 37°C for 1 h before assembling each pad. The NC membrane was pasted on the cardboard. The conjugated and absorbent pads were also pasted on the cardboard and overlapping with each side of NC membrane about 2 mm. The sample pad was laid over (2 mm) the absorbent pad and pasted on the cardboard as well. AGISMART RP-1000 (rapid test immno-strip printer) (REGA biotechnology Inc.) was used to dispense capture mAb (500 µg/mL) on the test zone and rabbit anti-mouse IgG antibody (500 µg/mL) (REGA biotechnology Inc.) on the control zone on the NC membrane. The distance between 2 zones was 5 mm. The strips were prepared and assembled at the low humidity environment, packaged in the aluminum pouch, and stored at 4°C until used.

### Test procedure and cross-reactivity test

A frozen muscle sample (0.03 g) was put into 1.5 mL centrifuge tube and homogenized with 1 mL PBS (containing 0.1% BSA) by bamboo stick. Five hundred µL supernatant was collected and transferred to a new 1.5 mL centrifuge tube. The result can be observed directly after soaking the sample pad of the strip into the supernatant for 5−10 min. Twenty-three different muscle samples (including 15 species of cetaceans and 8 species of other animals) were tested in triplicate. This process was repeated 5 times by 5 different inspectors.

## Results

### Muscle extracts

**Figure 2:**
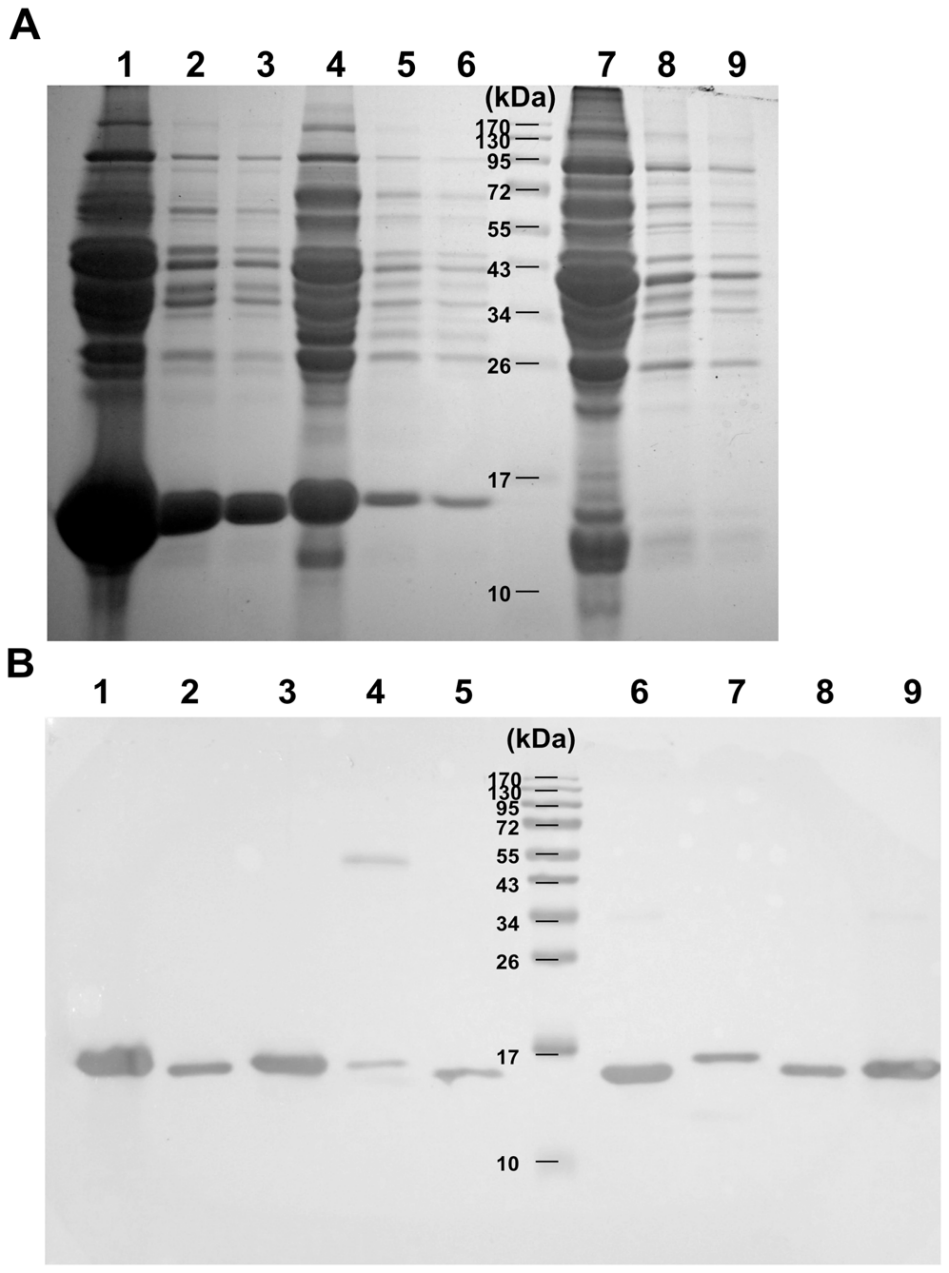
SDS-PAGE and western blot analysis of muscle extracts of different species. A: Lane 1−3: dwarf sperm whale at no, 1:10 and 1:20 dilution. Lane 4−6 and lane 7−9: cow and rat at the same dilution series, respectively. B: western blot analysis using commercial rabbit anti-human myoglobin polyclonal antibody. Lane 1−9: cow, goat, pig, rabbit, rat, dwarf sperm whale, minke whale, bottlenose dolphin and finless porpoise, respectively.

The muscle extracts of different animal species containing soluble proteins were separated by SDS-PAGE (Fig. 2A). The proteins of the predicted size bands (∼17 kDa) for Mb were found in four species (rat, cow, and dwarf sperm whale *Kogia sima*). Dwarf sperm whale contained the most abundant Mb than the others did. According to the result of SDS-PAGE, the 1∶20 dilution of dwarf sperm whale was too high to be used in western blot. For showing better western blot result, 1∶50 dilution of cetacean and seal and 1∶5 dilution of cow and rat extracts were used. The antigenic protein banding patterns of approximate 17 kDa were observed in all species in western blot using commercial rabbit polyclonal antibody against human-origin Mb (Fig. 2B). Thus the muscle extracts using our extraction protocol clearly contain Mb confirmed by SDS-PAGE and western blot.

### Peptide design

**Figure 3:**
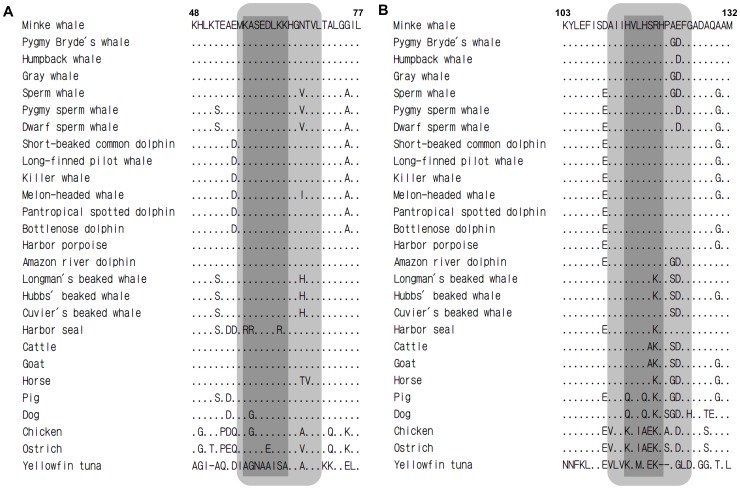
Amino acid sequence identity between cetaceans and other animals in 2 antigenic regions of myoglobin: (A) region 2, (B) region 4. Dark gray column: core antigenic region. Light gray column: peptide design region.

The conserved fragment in cetaceans and artiodactyls (KASEDLKKH) including the antigenic reactive region 2 was found (Fig. 3A). The sequence (HVLHSRHPA) including the region 4 was conserved in cetaceans except 2 beaked whales (Fig. 3B). The hydrophobic amino acids were added to the N-terminal of core antigenic region for decreasing the risk of discomposing in the immunoreactions, and the C-terminal of core antigenic region was lengthened for exposing the antigenic reactive regions to immunocytes. The amino acids were added according to the sequence identity. Furthermore, cysteine (Cys, C) was added to the C-terminal for facilitating the conjugation with a carrier protein (OVA). In sum, 2 peptides were designed based on the Mb antigenic reactive regions, and synthesized as the haptenic antigens: general peptide (GP, MKASEDLKKHGNTVLC) and specific peptide (SP, AIIHVLHSRHPAEFGC). Methionine (Met, M), alanine (Ala, A), and isoleucine (Ile, I) are hydrophobic amino acids. The mAb raised by GP was predicted to bind Mb of cetaceans and artiodactyls but not seal. The mAb raised by SP was predicted to bind only Mb of cetaceans.

### Monoclonal antibody characteristics

**Figure 4:**
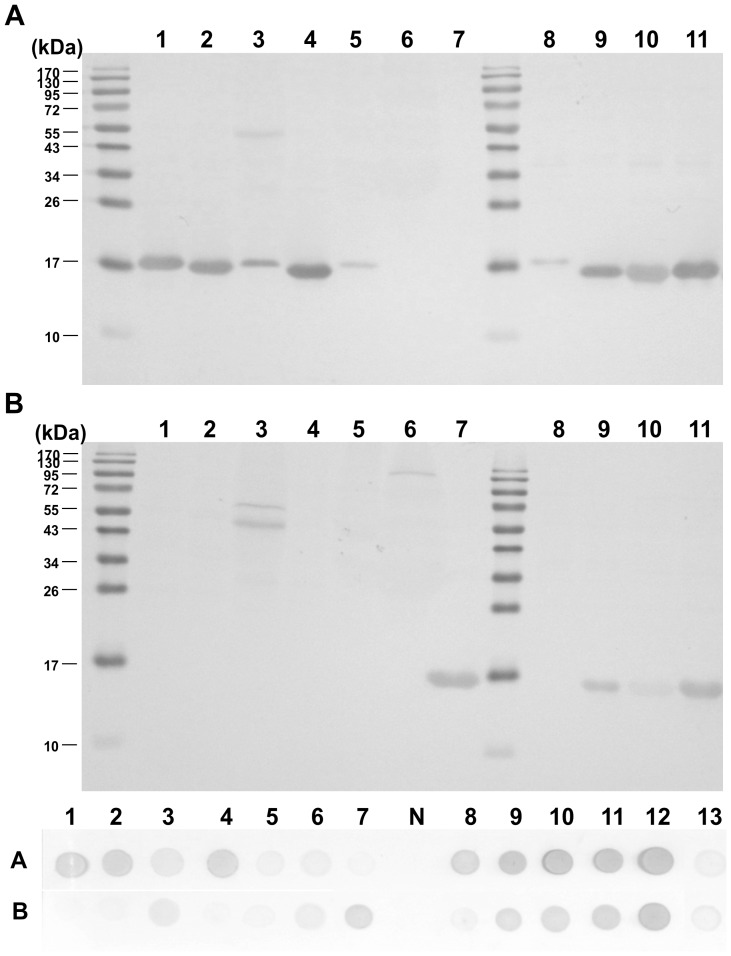
Western blot and dot blot analysis using the hybridoma supernatants. (A) CGF5H9, (B) CSF1H13. 1−7: cow, goat, pig, dog, rabbit, tuna and seal, respectively. 8−13: minke whale, bottlenose dolphin, dwarf sperm whale, finless porpoise, pygmy killer whale and chicken, respectively. N: PBS (negative control).

Two IgG_1_ mAbs, CGF5H9 (raised by GP) and CSF1H13 (raised by SP) were selected based on the results of western blot and dot blot (Fig. 4). In western blot analysis of CGF5H9, a single main band of approximate 17 kDa was detected in cetaceans/cow/goat/pig/dog/rabbit, and it showed no cross-reactivity with tuna/chicken/seal while there was a non-specific signal in pig. Common minke whale (*Balaenoptera acutorostrata*) showed comparatively weaker signal than other cetaceans did. CSF1H13 detected a band of approximate 17 kDa only in cetaceans/seal and it presented no cross-reactivity with any of other species while there were some non-specific signals in pig and tuna. Minke whale showed no signal in 1∶25 dilution but strong signal in 1∶10 dilution (data not shown). Identical results were obtained in dot blot.

**Figure 5:**
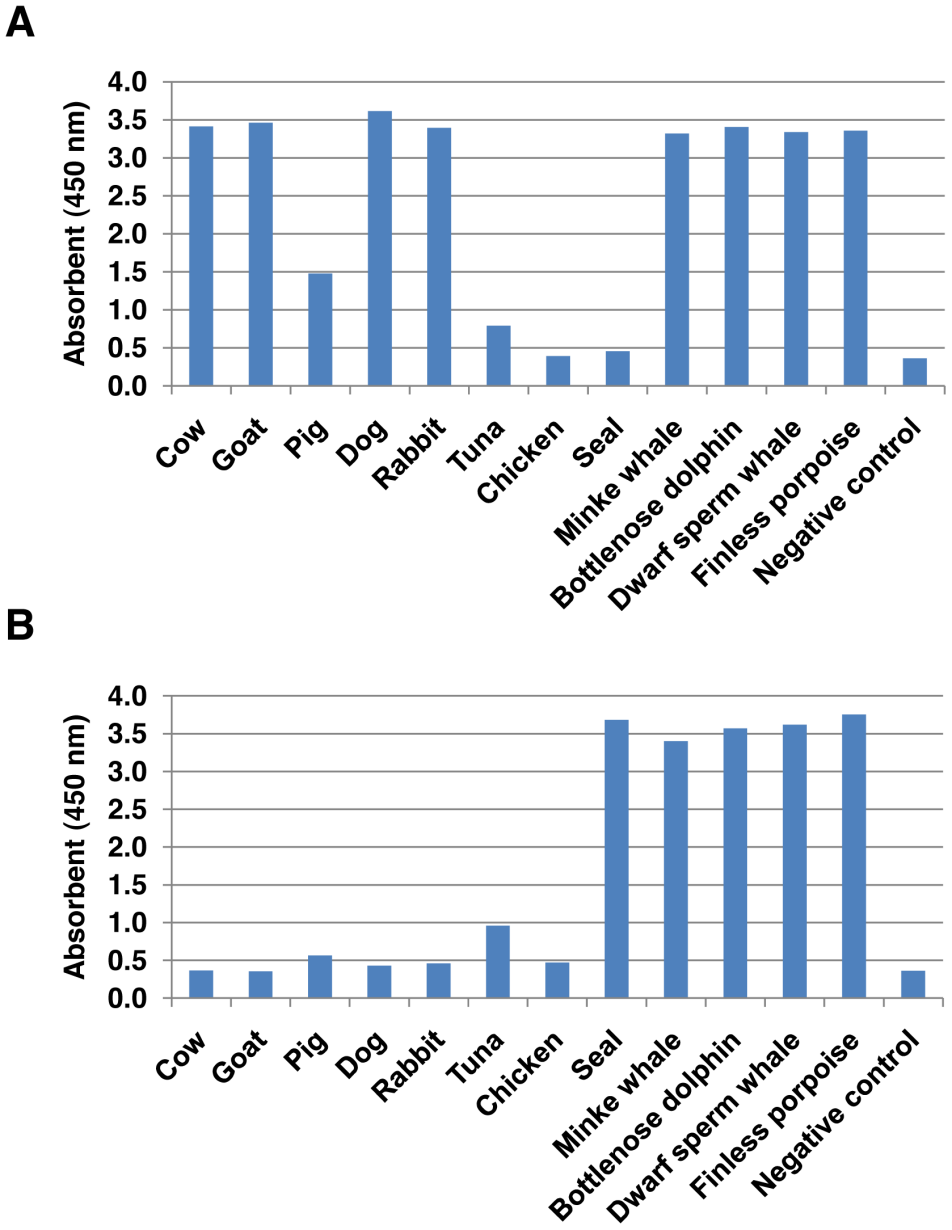
Indirect ELISA of muscle extracts of different species using purified mAbs: (A) CGF5H9, (B) CSF1H13.

The result of indirect ELISA showed the quantification of cross-reactivity of the mAbs toward cow, goat, pig, dog, rabbit, chicken, tuna, and other marine mammals (Fig. 5). CGF5H9 expressed strong reactivity toward cetaceans/cow/goat/dog/rabbit (OD value > 3.0) and weak reactivity toward pig (OD value  =  1.5). It had no reactivity toward seal, chicken and tuna. CSF1H13 showed strong reactivity only toward cetaceans and seal (OD value > 3.0). Both CGF5H9 and CSF1H13 gave similar strong reactivity toward 4 different cetacean species from different families (Balaenopteridae, Delphinidae, Kogiidae and Phocoenidae).

### Test procedure and cross-reactivity test

After the strip was soaked into the muscle extract, the colloid gold-labeled CGF5H9 would be dissolved and traveled together to the absorbent pad. When the extract was from a cetacean species, the colloid gold-labeled CGF5H9 bound with the Mb that was subsequently trapped by the mAb CSF1H13, and then a red line was observed at the test zone. Colloid gold-labeled CGF5H9 would then be trapped by the rabbit anti-mouse IgG antibody in the control zone whether the extract was from cetaceans or not. In sum, 2 red lines were presented on the strip when the sample contained the Mb of cetaceans. There was only 1 red line (control line) appeared on the strip when the sample contained no Mb of cetaceans.

**Figure 6:**
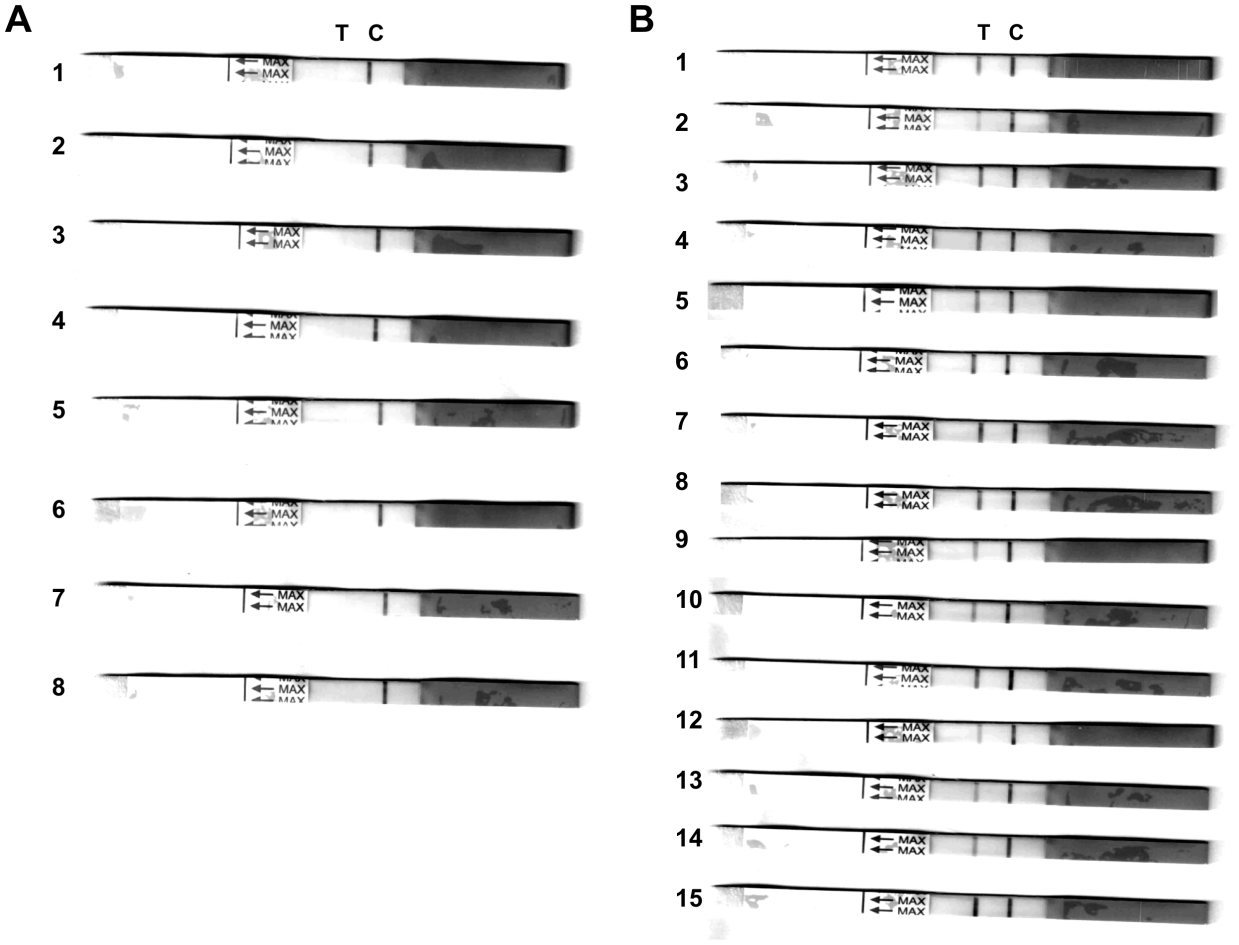
Specificity of the immune colloidal gold strip. T: test line. C: control line. (A) Non-cetacean samples: 1: Cow. 2: Goat. 3: Pig. 4: Dog. 5: Rabbit. 6: Tuna. 7: Chicken. 8: Harbor seal (*Phoca vitulina*). (B) Cetacean samples: 1: Common minke whale (*Balaenoptera acutorostrata*). 2: Omura’s whale (*Balaenoptera omurai*). 3: Bottlenose dolphin (*Tursiops aduncus*). 4: Bottlenose dolphin (*T. tuncatus*). 5: Fraser’s dolphin (*Lagenodelphis hosei*). 6: Indo-Pacific humpback dolphin (*Sousa chinensis*). 7: Risso’s dolphin (*Grampus griseus*). 8: Pantropical spotted dolphin (*Stenella attenuata*). 9: Rough-toothed dolphin (*Steno bredanensis*). 10: Pygmy killer whale (*Feresa attenuata*). 11: Short-finned pilot whale (*Globicephala macrorhynchus*). 12: Melon-headed whale (*Peponocephala electra*). 13: Dwarf sperm whale (*Kogia sima*). 14: Pygmy sperm whale (*K. breviceps*). 15: Finless porpoise (*Neophocaena phocaenoides*).

The muscle extracts of 23 species were tested in triplicate, and this process was repeated 5 times by 5 different operators. The control line showed the red signal obviously in all tests, and the background color of all strips was clear. No red line was revealed in the test zone for the samples from cow, goat, pig, dog, rabbit, tuna, chicken, and seal. Moderate to strong red signal were observed in the test zone for the samples from cetaceans (Fig 6). The specificity and the sensitivity of this strip are 100%.

## Discussion

In this study we developed 2 mAbs against synthetic peptides of Mb. Since the synthetic peptides present specific epitopes, screening suitable hybridoma cell clones would be more efficient. Kao and Hodges [23] reported the antibody titers against the native protein of the mice immunized with the synthetic peptide-conjugate were higher than the titers of mice with the native protein, and the affinities of anti-peptide sera for the intact target domain were significantly higher than those of anti-protein sera. These results showed significant advantages in using a synthetic peptide-conjugate over its cognate protein. Moreover, it is likely that the 2 mAbs in this study would not inhibit each other’s binding to the antigen because these 2 mAbs were developed using epitopes with known relative locations. Therefore the following sandwich-based immunoassay that is the most commonly used format in commercial kits [24] could be applied. In contrast, it is needed to do epitope comparison by performing additivity test for identifying a suitable pair of mAbs before designing a sandwich-based immunoassay when the mAbs are raised from purified protein or crude protein extract and the information of epitopes is unknown.

These 2 mAbs have different characteristics. The mAb CGF5H9 has moderate to strong affinity toward cetaceans, cow, goat, pig, dog, and rabbit, and no affinity toward seal, chicken and tuna, which was predicted based on the sequence identity. Atassi [20] has suggested the side-chain properties of amino acid sequence of Mb might affect the epitope structure. Each amino acid has specific properties due to its side-chain, and the structure of a protein is determined by the composition of its amino acids [25]. Consequently differences in amino acid properties might contribute to structure changes. The sequence of seal has 3 different amino acids in core antigenic region 2: one is a polarized residue (Arg, R) in place of the uncharged one (Ala, A) and the other two (Arg, R) are in the same group (polar basic) with the original residue (Lys, K). Thus the difference of amino acid residues between seal and cetaceans leads to the affinity change of antibody due to different epitope structures. Moreover, we increased the length of the synthetic peptides (immunogen) at the C-terminal region for assuring the core antigenic region being recognized and the difference between 2 different epitope sequences (cetaceans/seal) could be efficiently differentiated. The logic of peptide design was supported by the result that CGF5H9 has definitely different affinities toward cetaceans and seal. Although chicken has an identical sequence with dog in the core antigenic region 2, CGF5H9 has different affinities toward chicken and dog. It is supposed that the sequence difference in outer region might contribute to the structure change and consequent failure of antigen-antibody binding.

CSF1H13 showed strong affinity only toward cetaceans and seal. In core antigenic region 4 of seal a polarized basic residue (Lys, K) replaces the residue (Arg, R) in the same group. It likely causes minor epitope structure change and therefore seal has as strong reaction with CSF1H13 as cetaceans do. Because the core antigenic region sequences of seal and beaked whales are identical, CSF1H13 is supposed to have good affinity toward beaked whales as seal although no muscle samples of beaked whales are available in this study. CSF1H13 presented no reactivity toward cow and goat although they have only one-amino acid difference from seal in core antigenic region 4. It could be explained by the structure change due to the replacement of the uncharged residue (Ser, S) by a nonpolarized one (Ala, A). CSF1H13 showed strong reactivity toward minke whale in the indirect ELISA (1∶25 dilution) and in western blot and dot blot (1∶10 dilution), indicating that CSF1H13 can recognize minke whale in immunoblotting although higher Mb concentration is needed. There are 2 possible explanations for this phenomenon. First, different sample collection positions could result in different concentrations of Mb. For example, the content of Mb in swimming muscles (axial muscles) is significantly higher than in the non-swimming muscles in Fraser’s dolphins (*Lagenodelphis hosei*), spinner dolphins (*Stenella longirostris*) and a pygmy killer whale (*Feresa attenuata*) [15]. Second, the concentration of Mb would increase though life [26], and this whale might be at a young age. The minke whale was found in pieces from confiscation, and therefore the information of the sample collection position and age of this animal is unavailable.

We used CGF5H9 and CSF1H13 to develop a sandwich-based immune strip for the rapid differentiation of cetacean meat. The strip based on the sandwich format detects the antigen between 2 layers of antibodies. The colloid gold-labeled CGF5H9 recognizing the Mb of cetaceans, cow, goat, pig, dog, and rabbit was used as the detecting antibody to bind as much of the antigen as possible. CSF1H13 coated on the test zone allows fine detection of small differences in antigen and then only captures the Mb of cetaceans and seal. Only cetacean Mb is capable of binding to both antibodies and presents positive signal while the Mb from other animals can bind only 1 of the antibodies and presents negative result. The synthetic peptides for raising the mAbs were designed based on Mb amino acid sequences identity of 18 species of cetaceans from Balaenopteridae, Eschrichtiidae, Physeteridae, Kogiidae, Ziphiidae, Iniidae, Delphinidae, and Phocoenidae and the tested cetacean species included Balaenopteridae, Kogiidae, Delphinidae, and Phocoenidae, indicating the broad-range whale meat detection ability of this strip. Although the strip cannot identify the species of cetacean like DNA-based assay does, the operators can quickly obtain the result in 5−10 min at investigative spot by simply homogenizing small amount of muscle samples with bamboo stick and soaking the strip into it. The immune strip can offer a guide as to which samples most deserve further species confirmation for financially constrained enforcement agencies. Among the meat samples used in this study, the color of seal meat is very similar with that of cetacean meat because seal meat also contains high concentration of Mb (about 40 mg/g) [9]. This strip showed the ability to discriminate between seal and cetacean meat correctly. Because seal meat could be fraudulently sold as cetacean meat, the ability would be important especially in Asian market.

Many factors of the sample preparation/condition may affect the strip test result. First, using pure water instead of the extraction buffer for sample preparation may cause aberrant result. The salt-soluble protein cannot be extracted adequately from meat by pure water [27]. The target antigen, Mb, is a salt-soluble protein in meat and therefore we chose PBS containing 0.1% BSA as the extraction buffer for this strip. Second, the appropriate extraction buffer: meat sample ratio contributes to successful strip result interpretation. The ratio 1∶0.03 (mL: g) could present much clearer background than 1∶0.3 did (data not shown), and the red lines of the control and test zones could be observed obviously. Third, all the muscle extracts of cetaceans and other animals showed positive results when the boiled and soy sauce-cured samples were used (data not shown). Curing by soy sauce may hydrolyze the protein, and the procedure of boiling will denature the protein. Therefore only the fresh muscle samples could be used in the strip test. Fourth, Mb concentration of test sample may affect the signal strength of strip. The test result of Omura’s whale (*Balaenoptera omurai*) showed weaker signal than those of the other cetaceans. It is explained that the meat sample was obtained from a stranded newborn. The Mb concentration of calf is lower than that of adults and it would increase though life [26]. Moreover, the Mb concentration in swimming muscles (axial muscles) is significantly higher than in the non-swimming muscles in cetaceans [15]. The further research on this strip test using newborn or calf samples of cetaceans is needed although the signal using Omura’s whale newborn was still strong enough for differentiating it from other non-cetacean meat.

The consumption of small cetaceans and the number of species has increased in some regions during the last two decades [1]. Cetacean species once considered only as ‘by-catch’ are now sold for human consumption, and this is likely to encourage a market based on direct hunting [28]. The identification of cetacean meat is essential for reducing illegal trade that is a major threat to the long-term survival of certain cetacean species. Cetacean meat is not traditionally consumable food for people in many countries such as Taiwan. However, especially in Asia, some diners have been misled by untrustworthy reports of the health benefits from the relatively rare seafood. On the contrary, people consuming cetacean meats may be at high risk of ingesting high concentration of pollutants [3] or zoonotic pathogens [5]. Dolphinpox [29] and *Brucella* species [30], for example, have been supposed to be potential health risks of Peruvians that handled or consumed small cetaceans. Other zoonotic pathogens such as *Erysipelothrix rhusiopathiae*, *Mycobacterium* spp., *Lacazia loboi*, *Toxoplasma gondii*, and St. Louis encephalitis virus have been found in cetaceans (reviewed in [31]). The health hazard of these zoonotic pathogens in cetaceans may have been underestimated, attributable to misdiagnoses and underreporting of both diseases and cetacean meat consumption. The installation of this effective identification method may help to improve the reporting of cetacean meat possessing, decrease the illegal consumption very likely and have implications not only for cetacean conservation but also for public health management.
